# Symptom Management in Chronic Heart Failure: Strategies and Behaviours From Patients’ Perspectives—A Scoping Review

**DOI:** 10.1111/hex.70667

**Published:** 2026-04-13

**Authors:** Severin Pietsch, Sascha Köpke, Christa Büker, Irene Müller

**Affiliations:** ^1^ Faculty of Medicine and University Hospital Cologne, Institute of Nursing Science, University of Cologne Cologne Germany; ^2^ Faculty of Health, Institute for Educational and Health‐Care Research in the Health Sector, Hochschule Bielefeld—University of Applied Sciences and Arts Bielefeld Germany; ^3^ Department of Social Work and Health University of Applied Sciences Vorarlberg (FHV) Dornbirn Austria

**Keywords:** chronic heart failure, daily life, Dodd's Symptom Management Model, health behaviour, non‐recommended behaviour, symptom management

## Abstract

**Introduction:**

Chronic heart failure (CHF) affects millions and burdens health systems through high morbidity and mortality. Guidelines emphasise the need for self‐management. Yet the specific strategies patients use to perceive, appraise, and respond to symptoms remain insufficiently described. Symptom management is a core component of self‐management and directly shapes illness trajectories and quality of life.

**Objectives:**

To identify and map behaviours and strategies of adults with CHF to manage symptoms in daily life.

**Methods:**

We conducted a scoping review of MEDLINE (PubMed), CINAHL (EBSCOhost), Livivo and Cochrane Library (2006–2024). Data were analysed using Mayring's qualitative content analysis. Categories were derived deductively from heart failure guidelines and Dodd's Symptom Management Model and inductively from included studies. The category system was aligned with Dodd's Symptom Management Model, which guided the structuring of findings into symptom experience, management strategies, and outcomes.

**Results:**

Thirty‐one reports (qualitative, quantitative, mixed methods) met the inclusion criteria. Patients deploy diverse strategies across five domains: medication management, symptom monitoring, adjustment of daily activities, fluid and weight management, and lifestyle modification. Social support is pivotal. Many patients deviate from recommendations to balance demands, gaining short‐term relief at the potential expense of long‐term control. Such deviations function as pragmatic coping and indicate unmet information and structural needs.

**Discussion:**

Patients’ behaviours are integral to symptom management and should inform context‐sensitive professional support and care. Involving family members and utilising digital tools could enhance monitoring, decision‐making, and quality of life. Future work should apply Dodd's Symptom Management Model to clarify links between symptom experience, strategies, and outcomes and to develop approaches feasible and compatible with daily life.

**Patient or Public Contribution:**

After completing the core scoping review work, we conducted a PPIE session with a person living with heart failure (NYHA III) to validate the category system, the presentation of results, and practice implications. The feedback enhanced interpretability (micro‐level strategies) and supported a destigmatising view of non‐recommended behaviours.

**Abbreviations**
CHF: chronic heart failure
NRB: non recommended behaviour
NYHA: New York Heart Association
PPIE: Patient and Public Involvement and Engagement

## 1 Background

Chronic heart failure (CHF) is among the most prevalent cardiovascular diseases globally, associated with significant morbidity and mortality [1, 2]. In 2017, approximately 64.3 million people worldwide were affected, placing CHF among the leading causes of global health burden [3]. Effective disease management is essential to enhance patients’ quality of life, decrease hospitalisation rates, and improve clinical outcomes [4]. Guidelines highlight the importance of self‐management, a broad construct that encompasses adherence to pharmacological therapy, dietary modifications, regular physical activity, and the monitoring of and appropriate response to symptoms [4, 5]. Within this broader framework, symptom management represents a clearly defined subcomponent that focuses specifically on how patients perceive, appraise and respond to symptoms in everyday life. It includes strategies and behaviours for recognising and addressing symptoms, along with the evaluation of outcomes that may influence subsequent symptom‐related decisions [6]. This distinction is essential: while self‐management encompasses a wide range of health‐promoting activities associated with living with a chronic condition, symptom management focuses specifically on dealing with disease‐related symptoms and the actions taken in response to them. Heart failure specialist nurses play a key role in this context, supporting patients by providing education, facilitating early symptom recognition and guiding daily decision‐making [7]. However, symptom management ultimately relies on how patients perceive, interpret and respond to symptoms in their everyday lives. To better understand these patient‐driven processes, therefore, the Symptom Management Model proposed by Dodd et al. [6] offers a robust theoretical foundation. It conceptualises symptom management as a dynamic process comprising the three dimensions: symptom experience, management strategies and outcomes. The model highlights that strategies can be either supportive or counterproductive depending on the symptom and its context, and that patients’ behavioural responses are shaped by prior experiences, emotional states and situational demands. This makes the model particularly relevant for CHF, where fluctuating symptom patterns such as breathlessness, fatigue and fluid retention require continuous appraisal and adjustment. Therefore, this model is used as the guiding analytical framework for this scoping review to systematically map the diverse patient‐reported strategies identified in the literature.

Symptom management is influenced by individual and psychosocial factors and represents a central aspect of coping with the illness [6]. It significantly influences patients’ quality of life and the course of the illness, as it determines whether individuals are able to detect symptoms early and respond appropriately. Despite the emphasis on self‐management in clinical guidelines [4, 5, 8], the embedded concept of symptom management is mostly only briefly mentioned and receives limited elaboration. Previous studies have primarily focused on improving adherence through technological or educational interventions [9, 10], as well as on evaluating the effectiveness of treatment programmes aimed at supporting patients in managing chronic illness [11, 12]. However, there is a lack of research on the behaviours and strategies patients actually apply in everyday symptom management. To address this gap, a scoping review was conducted with the aim of providing a comprehensive overview of symptom management behaviours and strategies described in the literature among patients with CHF. A deeper understanding of these patient‐reported practices is essential for developing targeted interventions that are grounded in the lived experiences of patients with CHF.

## 2 Methods

This scoping review follows the methodological framework proposed by Arksey and O'Malley [13], supplemented by recommendations from the Joanna Briggs Institute (JBI) [14]. Reporting adheres to the PRISMA‐ScR checklist [15]. The study protocol was registered in the Open Science Framework (OSF—osf.io/2p867).

### 2.1 Identification of the Research Question

The study design follows a scoping review approach that explores strategies and behaviours in the symptom management of patients with CHF. The research question was developed using the PCC (Population, Concept, Context) framework to ensure a systematic and transparent analytical process (see Table 1).

**Table 1:** PCC framework.

Population	Patients with chronic heart failure, NYHA Classes II–IV
Concept	Strategies and behaviours in symptom management
Context	Everyday life

Abbreviation: NYHA, New York Heart Association.

The scoping review was guided by the following research question:

Which strategies and behaviour do patients with CHF (NYHA Classes II–IV) use in symptom management to implement pharmacological and non‐pharmacological treatment recommendations in their daily lives?

### 2.2 Identification of Relevant Studies

The literature search was conducted in MEDLINE via PubMed, CINAHL via EBSCOhost, Livivo, and Cochrane Library. The initial database searches were carried out between September and December 2023. To ensure that the evidence base remained current during the analysis phase, an automatic search alert was activated in MEDLINE via PubMed and was monitored until December 2024, and manual update searches using the original search strings were performed in CINAHL via EBSCOhost and Livivo during the same period. These update searches did not yield any additional eligible studies. No further relevant publications were identified between the final update in December 2024 and manuscript completion. Thus, the evidence base of this review reflects all studies available up to December 2024. In addition, backward citation tracking was performed. The search strategy and key search terms are summarised in Table 2, with the complete search string for PubMed provided as an example. To also capture studies in which symptom management is addressed as a sub‐component, the search was expanded to include the term ‘self‐management’ and related concepts.

**Table 2:** Search terms, controlled vocabulary (MeSH/MH) and database‐specific search string via PubMed.

Component (PCC‐framework)	Search terms (Free text)	MeSH‐/subject headings (Across all databases)
Population	Heart Failure, Cardiac Failure, CHF, Chronic Heart Failure, Congestive Heart Failure	***Heart Failure*** (MeSH, PubMed; MH, CINAHL; MeSH, Livivo; N/A, Cochrane)
Concept	Self Care, Self‐Care, Self‐Management, Self Management, Self‐Regulation, Self Monitoring, Self‐Monitoring, Self Care Strategies, Self Care Practices, Symptom Management, Symptom Control, Symptom Relief, Symptom Reduction, Symptom Improvement, Strategy, Strategies, Behavior, Behaviors, Behaviour, Behaviours, Help Seeking Behavior	***Self Care***, ***Health Behavior*** (MeSH, PubMed; MH, CINAHL; MeSH, Livivo; N/A, Cochrane); *Help Seeking Behavior* (MH, CINAHL)
Context	Daily Routine, Everyday Life, Daily Living, Daily Activities	***Activities of Daily Living*** (MeSH, PubMed; MH, CINAHL; MeSH, Livivo; N/A, Cochrane)
Search String (PubMed)		
((“self care”[MeSH Terms] OR “health behavior”[MeSH Terms] OR “self care”[All Fields] OR “behavior”[All Fields] OR “behaviors”[All Fields] OR “behaviour”[All Fields] OR “behaviours”[All Fields] OR “self monitoring”[All Fields] OR “self regulation”[All Fields] OR “self management”[All Fields] OR “symptom management”[All Fields] OR “strategy”[All Fields] OR “strategies”[All Fields]) AND (“heart failure”[MeSH Terms] OR “heart failure”[Title/Abstract] OR “cardiac failure”[Title/Abstract] OR “CHF”[Title/Abstract] OR “chronic heart failure”[Title/Abstract] OR “congestive heart failure”[Title/Abstract]) AND (“activities of daily living”[MeSH Terms] OR “daily routine”[All Fields] OR “everyday life”[All Fields] OR “daily living”[All Fields])) AND (2006:2024[pdat])		

Abbreviations: MeSH, Medical Subject Headings (PubMed, Livivo); MH, Major Heading (CINAHL); N/A, not applicable.

### 2.3 Study Selection

The inclusion and exclusion criteria ensured that the studies included in this review focused on symptom management from the patients’ perspective. The population was limited to patients with CHF in NYHA Classes II–IV, as no symptoms are typically present in Class I. Patients receiving palliative care or with a ventricular assist device were excluded because their care focuses on other aspects of symptom management. Studies were included if they investigated strategies and behaviours related to symptom management, even when this was only a subcomponent of a broader self‐management focus. Crucially, in studies featuring mixed samples (including NYHA I percentages), data extraction and coding were strictly confined to passages that explicitly reported symptom experiences and management strategies from symptomatic participants, thereby mitigating potential conceptual bias. Studies primarily targeting educational interventions or training programmes were excluded. Only studies examining symptom management in everyday life were considered eligible. In addition to qualitative studies, quantitative studies were included provided that they were based on patient self‐reports. The detailed inclusion and exclusion criteria are presented in Table 3. In line with established methodological guidance for scoping reviews [14], no formal critical appraisal was conducted, as the aim was to map the range and characteristics of existing evidence rather than to assess study quality.

**Table 3:** Eligibility criteria for study inclusion and exclusion.

Criterion	Inclusion criteria	Exclusion criteria
Population	Patients with chronic heart failure, NYHA Classes II–IV	NYHA Class I; palliative care; ventricular assist devices; cognitive impairments; < 18 years of age
Concept	Individual strategies and behaviours in symptom management	Participation in educational programmes or training interventions
Context	Everyday life, including occupational, social, and home environments	Clinical settings
Study design	Qualitative studies, mixed‐methods studies, quantitative studies based on patient self‐report	Studies not reporting primary patient‐reported data (e.g., proxy‐reported studies, observational studies without self‐report, reviews, commentaries).
Language	German, English	Other languages
Publication period	2006–2024; plus one relevant study from 2000 (identified via backward citation tracking)	—

Study selection was conducted using the review software Rayyan. Two independent reviewers performed the title and abstract screening. A pilot test with 100 records was conducted to ensure consistent application of the inclusion criteria. This pilot served as a formal calibration phase to ensure a shared understanding and application of the criteria. Since initial disagreements were minimal and swiftly resolved through consensus discussions, a third reviewer was not needed. The subsequent full text screening was performed completely by both reviewers.

### 2.4 Charting the Data and Collating, Summarising, and Reporting the Results

From the included studies, relevant data were extracted systematically using a pre‐tested standardised extraction protocol (title, authors, year, country, publication type, publication source, population, DOI, key findings). Subsequently, a qualitative content analysis according to Mayring [16] was conducted by using the top‐down–bottom‐up method to identify patterns and relevant findings in the context of symptom management in heart failure. This approach allows for the development of both deductive and inductive categories and supports a structured analysis of the extracted data [16]. The deductive categories (main and subcategories) were derived from heart failure guidelines [4, 5] and the position paper by Jaarsma et al. [8], representing the current standard of care. The Symptom Management Model by Dodd et al. [6] with its three core dimensions, served as the guiding conceptual framework to filter and validate the relevance of the guidelines’ statements and ensure they focused exclusively on symptom management from the patients’ perspective. These three dimensions conceptually cover:

- Symptom Experience (D1): The subjective perception and appraisal of symptoms.
- Management Strategies (D2): The behavioural, pharmacological and cognitive interventions initiated by the patient.
- Outcomes (D3): The consequences of these strategies across physical, functional and psychosocial domains.

Inductive categories were developed iteratively from the data in order to capture aspects not previously addressed. This approach allowed for a comprehensive and conceptually grounded synthesis while remaining open to patient‐reported novelty. The category development was reviewed by the second reviewer. Coding was validated on a sample basis and the codebook was cross‐checked to ensure consistency and reliability. To ensure analytical rigor and intercoder consistency (trustworthiness), the first studies were jointly coded as a calibration phase. Subsequently, a random sample of the included studies was independently cross‐coded by the second reviewer. All disagreements regarding category assignment were immediately resolved through frequent consensus meetings and the iterative refinement of the codebook. Furthermore, the entire category development was regularly audited and reflected upon during weekly project meetings to ensure conceptual validity in relation to the theoretical framework. This multi‐stage validation process ensured a transparent and traceable assignment of data. For the analysis, MAXQDA (Version 24, VERBI Software, Berlin, Germany) was used to support systematic coding and traceability. To visualise the findings and document the analytical process transparently, summary tables as well as the grid and memo functions of MAXQDA were utilised. The presentation of findings is based on a category system that reflects the core insights. Results are presented both narratively and the form of tables.

### 2.5 Patient and Public Involvement and Engagement (PPIE)

To strengthen interpretability and practice relevance, we conducted a retrospective PPIE consultation after completing the core review processes. One individual living with CHF (NYHA III) participated as an advisory partner. The discussion focused on (i) reviewing and validating the category system and presentation of results, (ii) clarifying the delineation of symptom management within the broader self‐management construct, and (iii) prioritising practice‐relevant implications, including family involvement, low‐threshold access to support and the potential role of heart‐failure specialist nurses. PPIE contributor were involved exclusively at the interpretation stage following completion of the core review processes. The contributor did not influence the search strategy, eligibility criteria, study selection, data extraction or synthesis.

## 3 Results

### 3.1 Study Selection Process

After removing duplicates in Rayyan, a total of *n* =  1700 references remained for title and abstract screening, of which 1629 were excluded. The full texts of 71 reports were assessed for eligibility.

Reports were excluded if they did not describe specific behaviours and strategies (*n* = 36), focused on relatives’ perspectives (*n* = 1), or represented secondary literature such as systematic reviews (*n* = 3). Ultimately, 31 reports representing 29 unique patient samples met the inclusion criteria. This is because, in two instances, two separate reports were derived from the same patient sample (i.e., four reports based on two samples). These reports were retained as they addressed distinct research questions and provided differentiated insights.

**Figure 1:**
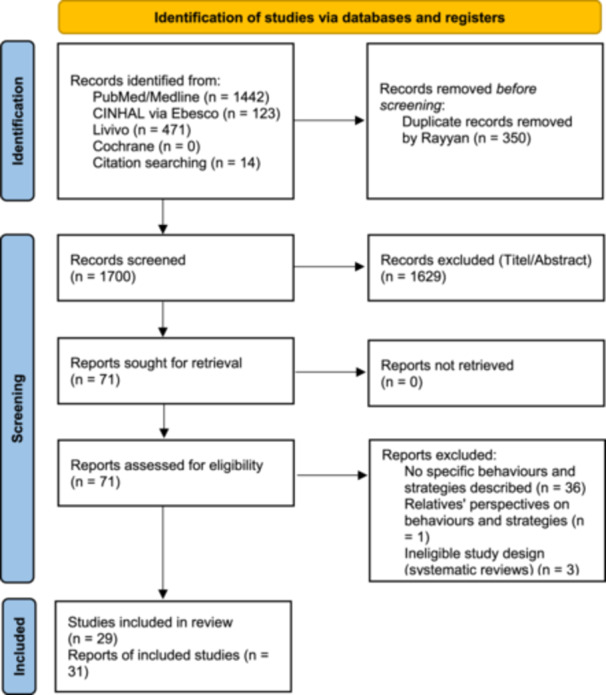
PRISMA flowchart illustrating the study selection process.

The complete selection process is illustrated in the PRISMA flowchart (see Figure 1). A list of excluded studies with reasons for exclusion is provided in the Supporting Information (Supporting Information S1: Table S1).

### 3.2 Characteristics of Included Studies

The included studies demonstrated considerable methodological and geographical heterogeneity. Most studies were conducted in the United States (*n* = 11), followed by Sweden (*n* = 3). Two studies each were conducted in Singapore, Iran, Taiwan and Canada. Single studies originated from Japan, Norway, Australia, Spain, Slovenia, the Netherlands, and the United Kingdom.

In terms of methodological approach, the review encompassed 19 qualitative studies, 9 quantitative studies, 3 mixed‐methods studies. Qualitative studies primarily utilised semi‐structured interviews and focus groups. Quantitative studies employed self‐report questionnaires (e.g., EHFScBS, SCHFI) and medical record analyses. Mixed‐methods studies combined both qualitative and quantitative approaches.

In total, the studies included *n* = 2114 participants across *n* = 29 samples. Gender was reported in *n* = 29 studies, with 937 women (44%) and 1177 men (56%). Age was documented in *n* = 29 samples, with a mean age of approximately 65 years and a range from 21 to 95 years (*n* = 18). NYHA classification was reported in *n* = 23 studies, with most participants classified as NYHA Class II or III. An overview of the included studies and their key characteristics is provided in Table 4.

**Table 4:** Overview of included studies with study characteristics and participant information.

Author(s), year	Country	Study design	Data collection methods	Data analysis methods	Sample (*n*; gender [F/M]; age [mean, range]; NYHA [class])
Bennett et al., 2000 [17]	USA	QS	Focus groups	Thematic analysis	*n* = 23; 7F/16M; 60 [N/A]; [N/A]
Chew and Lopez, 2018 [18]	Singapore	QS	Photovoice methodology	Thematic analysis	*n* = 16; 5F/11M; 61.5 [45–79]; [N/A]
Chew et al., 2019 [19]	Singapore	QS	Unstructured interviews	Thematic analysis	*n* = 17; 4F/13M; 56.1 [32–80]; I [39.4%], II [70.6% sic!]
Dickson, McCauley & Riegel, 2008 [20]	USA	MMS	Semi‐structured interviews and standardised instruments (e.g., SCHFI)	Thematic content analysis, descriptive and nonparametric statistics	*n* = 41;15F/26M; 49.2 [25–65]; II [41.5%], III [58.5%]
Dickson, Deatrick, & Riegel, 2008 [21]	USA	MMS	Semi‐structured interviews and standardised instruments (e.g., SCHFI, cognition tests)	Thematic content analysis, descriptive and nonparametric statistics	Same sample as Dickson et al. [20]
Falk et al., 2007 [22]	Sweden	QS	Semi‐structured interviews	Phenomenographic analysis	*n* = 17; 5F/12M; 72 [55–83]; III [*n* = 11], IV [*n* = 6]
Friedman et al., 2008 [23]	USA	QnS	Semi‐structured interviews, Preadmission Illness Behavior Questionnaire symptom checklist	Descriptive statistics (e.g., Chi‐square tests, *t*‐tests)	Total: *n* = 212; 103F/109M; 72.5 [N/A]; N/A CHF history group: *n* = 148; 76F/72M; N/A [N/A]; N/A No CHF history group: *n* = 64; 27F/37M; N/A [N/A]; N/A
Gary, 2006 [24]	USA	QS	Semi‐structured interviews	Thematic analysis	*n* = 32; 32F/0M; 68 [N/A]; II [41%], III [59%]
Gheiasi et al., 2023 [25]	Iran	QS	Semi‐structured interviews	Thematic analysis	*n* = 15; 7F/8M; 56.6 [N/A]; I [*n* = 1], II [*n* = 3], III [*n* = 11]
Giezeman et al., 2017 [26]	Sweden	QNS	Medical record reviews and questionnaire (e.g., EHFScBS‐9)	Descriptive statistics (e.g., Chi‐square tests, *t*‐tests, logistic regression)	*n* = 155; 66F/89M; 79 [N/A]; N/A
Hashimoto et al., 2023 [27]	Japan	QnS	Self‐administered questionnaires (e.g., EHFScBS, custom survey)	Logistic regression, descriptive statistics	*n* = 176; 58F/118M; 73 [63–81]; I/II [N/A], III/IV [30.7%]
Heo et al., 2021 [28]	USA	QS	Semi‐structured interviews	Content analysis	*n* = 20; 11F/9M; 51.9 [N/A]; II [25%], III/IV [75%]
Kaholokula et al., 2008 [29]	USA (Hawaii)	QS	Focus groups (e.g., with heart failure patients and caregivers, *n* = 4 groups)	Thematic analysis	Total: *n* = 36 (Subgroup: *n* = 11 CHF patients); 8F/3M; 55.3 [N/A]; N/A
Kamrani et al., 2014 [30]	Iran	QnS	Self‐administered questionnaire (e.g., EHFScBS, demographic survey)	Descriptive and inferential statistics (e.g., *t*‐tests, ANOVA)	*n* = 184; 113F/71M; N/A [60–95]; II [12%], III [47.8%], IV [40.2%]
Lee et al., 2015 [31]	USA	QnS	Self‐administered questionnaires (e.g., SCHFI)	Descriptive and inferential statistics (e.g., logistic regression)	*n* = 311; 110F/201M; 60 [N/A]; I/II [36%], III/IV [64%]
Li et al., 2019 [32]	Taiwan	QS	Semi‐structured interviews	Thematic analysis	*n* = 27; 10F/17M; 63 [34–79]; II [33%], III [52%], IV [15%]
Macabasco‐O'Connell et al., 2008 [33]	USA	QnS	Structured interviews and questionnaire (e.g., SCHFI)	Descriptive statistics and content analysis	*n* = 65; 36F/29M; 58.8 [N/A]; I [5%], II [35%], III [37%], IV [23%]
Mickelson and Holden, 2018 [34]	USA	QS	Audio/video recordings and written surveys	Qualitative content analysis	*n* = 61; 30F/31M; 73.31 [65–86]; II/III [100%]
Norberg et al., 2014 [35]	Sweden	QS	Semi‐structured interviews	Thematic qualitative content analysis	*n* = 10; 7F/3M; 81 [74–91]; II [*n* = 5], III [*n* = 4], IV [n = 1]
Nordfonn et al., 2020 [36]	Norway	QS	Semi‐structured interviews	Systematic text condensation (STC)	*n* = 17; 6F/11M; 62 [46–74]; II [*n* = 11], III [*n* = 6]
Reeder et al., 2015 [37]	USA	QS	Semi‐structured interviews	Thematic content analysis	*n* = 60; 27F/33M; 63 [25–88]; N/A
Riegel et al., 2010 [38]	Australia	MMS	Semi‐structured interviews, Self‐Care of Heart Failure Index (SCHFI), medical record reviews	Qualitative content analysis, Descriptive statistics	*n* = 27; 8F/19M; 68.7 [35–94]; II [59.3%], III [40.7%]
Santesmases‐Masana et al., 2019 [39]	Spain	QnS	European Heart Failure Self‐Care Behaviour Scale (EHFScBS), health literacy questionnaire	Statistical analysis using ANOVA tests	*n* = 318; 163F/155M; 77.9 [N/A]; I [*n* = 88], II [*n* = 163], III [*n* = 64], IV [*n* = 3]
Schnell‐Hoehn et al., 2009 [40]	Canada	QnS	Self‐care of Heart Failure Index (SCHFI), General Well‐being Schedule (GWBS)	Descriptive statistics, Pearson correlation, ANOVA	*n* = 65; 15F/50M; 59 [21–88]; I [8%], II [40%], III [44%], IV [8%]
Schumacher et al., 2018 [41]	Canada	QS	Semi‐structured interviews	Grounded theory methodology	*n* = 18; 11F/7M; 74.27 [37–88]; II [*n* = 6], III [*n* = 10], IV [*n* = 2]
Sedlar et al., 2021 [42]	Slovenia	MMS	Semi‐structured interviews, European Self‐Care Behaviour Scale (EHFScBS‐9),	Descriptive statistics (SPSS), qualitative content analysis	*n* = 80; 34F/46M; 72 [N/A]; II/III [100%]
Tung et al., 2012 [43]	Taiwan	QnS	Self‐Care of Heart Failure Index (SCHFI‐V6), demographic questionnaire	Descriptive statistics, Pearson correlation, multiple regression analysis	*n* = 86; 23F/63M; 65.7 [N/A]; II [*n* = 65], III [*n* = 21]
van der Wal et al., 2010 [44]	Netherlands	QS	Semi‐structured interviews	Thematic content analysis	*n* = 15; 6F/9M; 70 [42–87]; II [*n* = 7], III [*n* = 7], IV [n = 1]
Walthall et al., 2017 [45]	UK	QS	Semi‐structured interviews	Thematic analysis	*n* = 25; 10F/15M; 72.66 [53–86]; II [*n* = 2], III [*n* = 6], IV [*n* = 17]
Woda et al., 2015 [46]	USA	QS	Photovoice methodology, SCHFI questionnaire	Thematic content analysis	*n* = 10; 7F/3M; 67.5 [N/A]; I [*n* = 3], II [*n* = 4], IV [*n* = 3]
Woda et al., 2015 [47]	USA	QS	Photovoice methodology	Thematic content analysis	Same sample as Woda et al., 2015 [46]

*Note:* NYHA classifications were reported as presented in the original studies. Where studies provided proportions, percentages are reported; where absolute numbers were available, these are presented accordingly. No further standardisation was applied due to heterogeneous reporting formats across studies.

Abbreviations: F, female; M, male; MMS, mixed methods study; N/A, not available; NYHA, New York Heart Association; QnS, quantitative study; QS, qualitative study.

### 3.3 Synthesis and Thematic Mapping

The qualitative content analysis resulted in a structured category system capturing the strategies and behaviours reported across included studies. The system comprised deductive main categories and their associated subcategories, such as medication management, symptom monitoring, adjustment of daily activities, fluid and weight management, and lifestyle modifications. Inductively derived findings included the inductive main category ‘Factors contributing to non‐recommended behaviours’ (NRBs) and the Inductive Subcategories assistive tools, social support, and NRB. For the purpose of a conceptually coherent synthesis, the entire category system was subsequently mapped onto the three dimensions of Dodd's Symptom Management Model [6]: (D1) symptom experience, (D2) management strategies and (D3) outcomes. This restructuring preserves the full breadth of the original category system while aligning it with a theoretically grounded framework.

**Figure 2:**
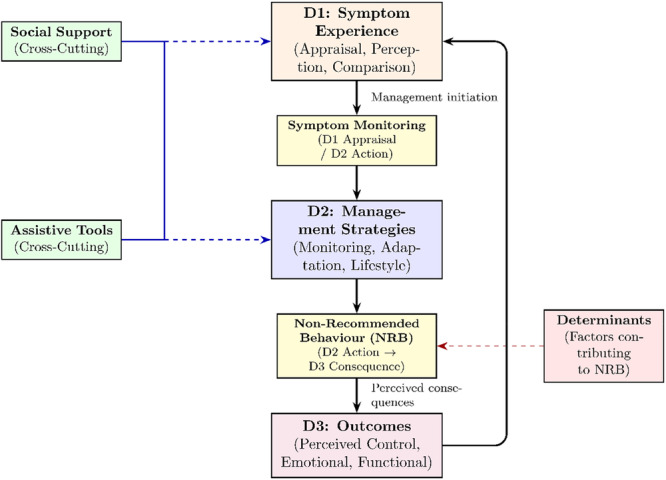
Synthesised symptom management cycle in chronic heart failure based on Dodd's Symptom Management Model (D1–D3). *Note:* The figure illustrates the dynamic interplay between symptom experience (D1), management strategies (D2), and perceived outcomes (D3). Symptom monitoring and non‐recommended behaviour represent dual‐function elements bridging adjacent dimensions, while specific determinants were identified as key drivers of non‐recommended behaviour. Social support and assistive tools function as cross‐cutting enablers influencing appraisal and management processes. Arrows indicate analytical relationships derived from the qualitative synthesis rather than causal or temporal pathways; although further reciprocal relationships between model dimensions are conceptually plausible, the available data did not allow these to be modelled explicitly within the present synthesis.

In this context, the three inductive subcategories required specific analytical consideration due to their cross‐cutting nature. They were identified as elements whose content was consistently present across all five deductive main categories. They were synthesised analytically to reflect their systemic function: Assistive Tools and Social Support were classified as cross‐cutting enablers. They modulate the entire management cycle by influencing both symptom appraisal (D1) and the execution of management strategies (D2). Furthermore, the category Symptom Monitoring was assigned a dual function (D1/D2), as it represents both the initial appraisal of symptoms and the active observational strategies employed. While the other deductive categories primarily reflect management strategies (D2), NRB was analysed using its dual function. This approach was necessary because NRB reflects both the behaviour itself as a situationally rational coping response and its resulting perceived impacts (positive or negative). The comprehensive model synthesised from the data, which visualises the structure and relationships of the identified core categories within Dodd's framework, is presented in Figure 2. A detailed mapping of all categories to the three dimensions is presented in Table 5 (Synthesis and Mapping of the Category System to Dodd's Model).

**Table 5:** Synthesis and mapping of the category system to Dodd's model.

Category (Hierarchy and name)	Type of derivation (Mayring)	Assigned Dodd's dimension	Rationale for analytical mapping (Core function)
I. Symptom Monitoring	Deductive MC	D1/D2	Subjective symptom appraisal (D1) combined with active observation and adaptation strategies (D2).
→ Help‐Seeking Behaviour	Deductive SC	D1/D2	Primarily rooted in symptom appraisal (D1), help‐seeking reflects an interpretive cognitive and emotional process that may translate into management actions (D2), such as seeking professional or informal support.
→ Behaviour for Specific Symptoms	Inductive SC	D2	Targeted actions for immediate physical symptom relief (e.g., dyspnoea, pain management).
II. Adjustment of Daily Activities	Deductive MC	D2	Behavioural modification focused on reduction of physical exertion and incorporation of rest breaks.
→ Activities outside the home	Deductive SC	D2	Strategic planning and adaptation for activities outside the home demands.
III. Medication Management	Deductive MC	D2	Strategies for adherence, routines, use of reminders, and management of challenges.
IV. Fluid and Weight Management	Deductive MC	D2	Strategies for monitoring body weight, fluid intake, and active regulation of diuretics.
V. Lifestyle Modification	Deductive MC	D2	Behavioural changes aimed at acceptance, diet, and management of physical activity/stress.
VI. Factors Contributing to Non Recommended Behaviour	Inductive MC	D2 (contextual challenges)	Complex interplay of individual, emotional, social, and lifestyle‐related factors that contribute to the emergence of non‐recommended behaviour.
Cross‐Cutting Subcategories	—	D1/D2/D3	Categories that operate across multiple dimensions and were synthesised separately due to their systemic function.
→ Assistive Tools	Inductive SC	D1/D2	Active utilisation (D2 Action) enable optimisation of management strategies (e.g., adherence, error reduction) and systematic data generation for symptom appraisal (D1).
→ Social Support	Inductive SC	D1/D2	Active engagement of social network (D2 Action) to facilitate the full spectrum of management strategies and validate subjective perception and assess urgency (D1).
→ Non‐Recommended Behaviour	Inductive SC	D2/D3	Actions and deviations (e.g., self‐adjusting treatment (D2), delayed response) that result in perceived consequences (positive or negative) (D3).

*Note:* Hierarchy abbreviations are MC (Main Category) and SC (Subcategory). Dodd's Model dimensions are abbreviated as D1 (Symptom Experience), D2 (Management Strategies), and D3 (Outcomes). The assignment to multiple dimensions (e.g., D1/D2, D2/D3) reflects the category's dual function within the theoretical framework.

The following sections present the results according to the three dimensions, synthesising how individuals with CHF perceive symptoms, act upon them, and experience the consequences of their symptom‐related decisions in everyday life.

#### 3.3.1 Symptom Experience (First Dimension of Dodd's Model)

Patients with CHF describe symptom experience as a continuous interpretive process shaped by bodily sensations, comparisons with previous episodes, and situational appraisal. They systematically observe physical changes, such as weight fluctuations and breathlessness, using these perceptions as indicators of potential deterioration [17, 34, 35, 41]. These observations are often embedded in daily routines; patients monitor signs through both objective markers (e.g., body weight, oedema) and subjective cues, including fatigue, reduced walking distance, or changes in physical performance [21, 35, 41, 42]. These interpretive processes are supported by both the use of assistive tools (e.g., for objective data capture such as weight and oedema) [42, 44] and the involvement of social resources for external validation of symptom assessment and urgency [17, 23, 36, 43]. A recurrent aspect of this process is the comparison between current sensations and past illness experiences [35, 41, 42]. Patients report distinguishing familiar symptoms from novel or unfamiliar sensations, which assists in assessing the urgency of the situation [28]. This comparative approach supports the identification of worsening symptoms and helps determine whether further action may be required [28, 41].

Symptom assessment is closely linked to perceived severity. Patients evaluate how threatening or manageable symptoms appear in the moment, and these evaluations influence subsequent actions [26, 31, 41]. Some patients describe uncertainty when interpreting symptoms, in these situations, they often consult family members to support appraisal and confirm their interpretation [17, 23, 36, 43]. This social involvement contributes to collaborative appraisal and helps reduce ambiguity surrounding symptom meaning [22, 23, 28, 36].

Breathlessness, fatigue, and sleep disturbances represent central components of the symptom experience in CHF. Breathlessness is frequently appraised through changes in posture, physical exertion, or the effectiveness of relief strategies such as positional adjustments, oxygen use, or reduced salt and fluid intake [17, 28, 35, 41, 42]. Fatigue is evaluated based on fluctuations in energy and the impact on daily performance, and it is often accompanied by sleep problems, which patients recognise through night‐time awakenings, difficulty falling asleep, or the need to change sleeping position [17, 28, 35, 41].

Emotional responses, including fear of deterioration or hope for spontaneous improvement, also contribute to how symptoms are perceived and evaluated [31]. Although emotional processes shape appraisal, patients primarily describe them as influencing the experience of symptoms rather than as determinants of behaviour. Following symptom appraisal, the individual determines the appropriate initial response, which marks the transition to management actions. This response ranges from continued observation and appraisal to the critical decision of whether and when to seek external support [26, 27]. Help‐seeking intentions are linked to the perceived severity and the individual's assessment of urgency [26, 41]. Patients may prefer watchful waiting and active monitoring over a defined period before escalating their actions [19, 21, 28, 46]. This decision‐making process highlights the core of this dimension (D1), as it reflects the immediate cognitive and behavioural result of the symptom experience before the full implementation of management strategies (D2) begins.

Across the included studies, symptom experience in CHF was described as an ongoing process rather than a static observation. This involved patients continuously monitoring bodily changes, comparing current sensations with prior episodes and evaluating the perceived severity of symptoms. Family members often contributed to interpreting these changes, particularly when uncertainty arose. Patients rely on these interpretive processes to determine whether symptoms require self‐initiated management or external assistance.

#### 3.3.2 Management Strategies (Second Dimension of Dodd's Model)

Management strategies among patients with CHF encompass a broad repertoire of behavioural adaptations aiming at controlling symptoms, preventing deterioration, and maintaining daily functioning. These strategies correspond to the model's second dimension, actions undertaken in response to perceived symptoms, and reflect both deliberate, structured approaches and situational, adaptive behaviours. Across the included studies, four major clusters of strategies emerge: (1) monitoring‐driven responses, (2) adaptation of daily activities, (3) regulation of fluid balance and weight, and (4) lifestyle adjustments. Assistive tools and social support function as cross‐cutting elements that reinforce or enable these strategies. Social support operates across all of these domains as an enabling strategy that assists patients in interpreting symptoms, implementing behavioural adjustments and maintaining day‐to‐day functioning.

##### 3.3.2.1 Monitoring‐Driven Responses

Patients with CHF act upon symptoms in various ways after identifying bodily changes. This relies on patients continually employing their symptom monitoring routines as an active management strategy to rapidly identify symptomatic deviations and trigger necessary management actions. These actions include adjusting posture, reducing physical activity, or modifying diet and fluid intake in response to breathlessness [17, 28, 35, 41, 42]. They engage in short‐term strategies such as resting in a quiet environment or applying breathing techniques to alleviate dyspnoea [35, 45]. For fatigue and sleep disturbances, patients reduce activity levels, increase rest, modify sleeping positions like using adjustable beds or employ calming routines such as reading or drinking herbal tea [17, 28, 35, 41].

Medication adjustments also form part of monitoring‐driven responses. Patients use diuretics or other medications in reaction to perceived fluid retention or worsening symptoms, sometimes after a brief period of observation [19, 21, 28, 46]. These actions are guided by perceptions of urgency and prior experience with symptom fluctuations [28, 41]. Help‐seeking is another core element of monitoring‐based responses. Some patients seek immediate professional support when symptoms appear severe or unfamiliar, while others adopt a watchful waiting approach [26, 31, 39]. Decisions are shaped by symptom severity, perceived threat, emotional responses, and the outcomes of previous attempts at self‐management [26, 31, 41]. If self‐initiated measures are perceived as insufficient, escalation to professional help becomes part of the symptom‐related decision process [37]. Social support contributes significantly to these decisions, particularly through family members who act as critical external resources. They are actively involved in assisting with symptom interpretation, affirming the perceived urgency, and initiating or encouraging contact with healthcare professionals when necessary [17, 22, 23, 28, 36, 43].

##### 3.3.2.2 Adaptation of Daily Activities

Patients restructure daily routines to align physical demands with symptom‐related limitations [17, 19, 35]. They adjust or redistribute tasks to conserve energy, combining errands, preparing meals in advance, or setting priorities to avoid overload [22, 32, 35, 41]. Many reduce physical exertion by limiting activities to certain areas of their home or avoiding unnecessary movements [35, 41, 45]. Scheduling planned rest periods is a common strategy to manage fatigue and prevent exhaustion [17, 27, 41]. Outside the home, patients adapt their activities and occupational roles by planning breaks, bringing their own meals, or using flexible work schedules to integrate health needs into daily routines [20, 21]. Preparation for leaving the house includes route planning, selecting comfortable clothing, and anticipating rest opportunities [22, 24, 36]. For longer trips, patients carry medication lists, emergency supplies, or identify healthcare facilities in advance [34, 35, 45].

Assistive tools such as mobility aids (e.g., walking sticks, walkers, wheelchairs) [17, 35, 45], household aids (e.g., shoehorns, mechanical kitchen tools) [17, 22, 35], and home modifications such as handrails or lifts [35] help reduce strain, enhance safety, and support independence [42]. Digital and telemedical tools supplement these adaptations by enabling remote monitoring and timely adjustments to therapy [17, 32]. Social support facilitates many of these adaptations by sharing household responsibilities, assisting with physically demanding tasks, or accompanying patients during activities outside the home, thereby reducing physical strain and supporting participation in daily life [22, 35, 36, 41].

##### 3.3.2.3 Regulation of Fluid Balance and Weight

Fluid and weight management represent central components of symptom control. Many patients regulate fluid retention by adjusting diuretic doses according to symptoms or weight changes [17, 20, 21, 25, 28, 34, 38, 44], sometimes following individual action plans [21, 31, 44]. Fluid intake is intentionally restricted, typically 1.5–2 L per day [18, 27, 39], and smaller vessels or pre‐measured portions assist in monitoring consumption [22, 30, 32]. Food‐related fluid intake is also considered [22].

Weight monitoring is used to detect fluid shifts, though frequency varies [18, 24, 33, 39, 40, 43]. Weight gain is interpreted as an indicator of possible retention and prompts diuretic adjustments [28, 34, 38, 44]. Patients additionally monitor oedema, particularly in the legs and abdomen [21], and use supportive strategies such as elevating the legs or wearing compression stockings [28]. Professional help is sought in acute situations or when symptoms escalate despite self‐initiated measures [43].

Recording tools, including diaries, are used to document fluctuations in weight and daily symptoms [42, 44]. Reminders such as post‐its, alarms, or visual cues help maintain consistency in monitoring [44]. Photographs of oedema are used to document bodily changes, facilitating early recognition of fluid accumulation [30]. Social support plays an active role in fluid and weight management by helping patients monitor weight or oedema, supporting adherence to fluid restrictions, and offering guidance or encouragement during symptom fluctuations [17, 26, 27, 32, 38, 39, 44].

##### 3.3.2.4 Lifestyle Modification

Lifestyle modification includes behavioural adjustments intended to reduce symptom burden and support long‐term stability. Acceptance of the illness is described as a prerequisite for integrating these changes into daily life, often supported by motivational techniques such as goal setting or reward systems [17, 25, 28, 30, 35, 45]. Gradual adjustment of habitual routines assists in sustaining modifications in diet, activity, and emotional regulation [20, 35, 41]. Dietary strategies focus on reducing salt and fat intake, adjusting portion sizes, and avoiding foods perceived as detrimental [17, 25, 28, 29, 32, 44, 46]. Patients adopt alternative seasonings or monitor the salt content of foods [17, 25, 32, 44]. Physical activity is adapted to the level of symptom tolerance. Many patients prefer low‐intensity activities such as walking or light exercise [18, 26, 27, 33, 39, 40, 42, 43, 46], while others participate in structured fitness programmes [17]. Activity levels are adjusted to avoid overexertion, and pacing strategies are applied to maintain participation without eliciting or worsening symptoms [28, 29, 45].

Relaxation strategies, such as meditation, breathing exercises, massage, or listening to music, help patients to manage stress and maintain emotional stability [28, 29, 36]. Social activities such as spending time with family members or participating in leisure activities, function as stabilising practices that support adherence to lifestyle adjustments [22, 38, 47].

Social support plays an important enabling role across lifestyle behaviours. Family members and friends assist with diet adherence, exercise routines, and the organisation of daily activities [19, 30, 34, 36, 46]. Involvement in shared activities, such as walking or grocery shopping, reinforces these behavioural adaptations [35, 42]. Furthermore emotional encouragement from relatives or peers helps patients sustain lifestyle changes during periods of fluctuating symptoms or reduced physical capacity [28, 32, 36].

##### 3.3.2.5 Challenges and Determinants of Strategy Implementation

The implementation of recommended management strategies is often complicated by a complex interplay of personal, social, and emotional factors, which can lead to actions described as NRB. While these actions deviate from guidelines, they are often perceived by patients as necessary coping strategies to maintain quality of life or short‐term stability.

Cognitive and Educational Barriers: Adherence is challenged by individual deficits in knowledge and confidence. Specifically, a lack of confidence in one's ability to control the disease, limited knowledge about the illness, or distorted risk perception complicate necessary adjustments [18, 19, 21, 22, 26, 28, 33, 42, 44]. Furthermore, negative past experiences with the healthcare system can lead to distrust and delay seeking of professional help [24, 28].

Emotional and Psychosocial Factors: Emotional factors frequently impede the adoption of recommended behaviour. Adherence conflicts arise when recommendations clash with deeply ingrained habits, which, when emotionally intensified, may lead to symptom denial and delayed treatment [19, 21, 36, 38]. Fear of worsening health, decompensation, or hospitalisation acts as a powerful driver of avoidance behaviour, further complicating proactive symptom management [17, 19, 24, 28, 32, 34, 42, 45, 46]. Some patients actively ignore symptoms to mitigate psychological distress or emotional overload, resulting in a refusal to take necessary therapeutic actions [34, 36].

Pragmatic and Situational Demands: External and situational constraints influence adherence, particularly regarding medication and fluid balance. Patients sometimes independently adjust their medication regimen (increasing, reducing, or discontinuing doses) due to demands of daily life, occupational needs, or the goal of regulating side effects [17, 19, 20, 21, 24, 34]. The decision to adjust or discontinue diuretics, for example, is often driven by practical factors such as the consumption of high‐salt foods, travel necessities, or the unavailability of public restrooms [17, 20, 21, 24, 34].

Strategies Involving Deviation from Recommendations (Actions): Actions involving deviation resulting from the above factors are observed across various aspects of symptom management. Regarding fluid and salt management, patients may consume sweetened beverages without accounting them in their fluid intake, excessively reduce their fluid intake to avoid oedema, or increase it after high‐salt meals in the mistaken belief of flushing out the system [17, 19, 22, 44]. Taste preferences or stressful situations may lead to the neglect of salt reduction, sometimes resulting in the washing of salty foods or the consumption of specific items to neutralise the salt content [17, 20, 28, 44]. Similarly, concerning Activity and Monitoring, patients may either exceed their physical limits or, conversely, limit activities to conceal disease‐related limitations (e.g., driving a car to mask reduced walking speed) [17, 18, 19, 24, 26, 35, 40, 44, 45]. Many patients also neglect to monitor their weight or respond only when fluid retention has already occurred [24, 26, 39, 44]. These actions reflect behaviours where the immediate goal (comfort, relief from side effects, social participation) supersedes adherence to long‐term health recommendations.

The spectrum of management strategies and their underlying operational principles, including the cross‐cutting elements and determinants, is systematically summarised in Table 6 (Core Symptom Management Strategies Mapped to Dodd's Model).

**Table 6:** Core symptom‐management strategies mapped to Dodd's model.

Dodd's dimension	Category (Focus)	Core strategy bundle (Synthetic essence)	Key operational principle (Function in model and application)
Symptom Experience	Symptom Monitoring	Symptom Appraisal and Historical Comparison	Establish flexible monitoring routines and use subjective/objective signals in comparison with personal health history for the interpretation and evaluation of symptom status.
	Social Support (Cross‐Cutting)	External Validation and Interpretive Aid	Provides an external frame of reference and emotional confirmation to reduce ambiguity during symptom appraisal.
	Assistive Tools (Cross‐Cutting)	Data Generation for Appraisal	Provides objective measurement data (weight, edema) and documentation tools to enhance the systematic precision of symptom perception (appraisal).
Management Strategies	Symptom Monitoring	Symptom Monitoring and Action Triggering	Utilise continuous self‐observation and interpretation of symptom status to trigger deliberate timely actions and management responses.
	Adjustment of Daily Activities	Functional Adaptation and Energy Management	Reduce physical exertion, utilise rest breaks, and reorganise tasks/environment to maintain functional independence.
	Medication Management	Routine Integration and Treatment Consistency	Develop routines and use reminders (boxes, apps) to ensure reliable and regular intake.
	Fluid and Weight Management	Regulation and Fluid Balance Control	Actively monitor weight and fluid status and implement agreed diuretic adjustments as part of daily symptom management.
	Lifestyle Modification	Emotional Stabilisation and Health Behaviour Integration	Utilise emotional strategies (e.g. stress management) and acceptance for the long‐term integration of diet and physical activity.
	Assistive Tools (Cross‐Cutting)	Technology and Tool Utilisation	Actively use analogue/digital aids (mobility, documentation) for symptom control and independence.
	Social Support (Cross‐Cutting)	Social Network Utilisation and Collaboration	Engage family/peers for monitoring, delegation, emotional support, and to maintain social participation.
	Non‐Recommended Behaviour (Cross‐Cutting)	Preserving Autonomy via Situational Adjustment	Understand the deviation as a situationally rational coping strategy to maximise autonomy or quality of life.
	Factors Contributing to NRB	Contextual Barriers and Individual Determinants	Identify barriers (fear, time constraints, knowledge gaps) as direct triggers for NRB and offer targeted support.
Outcomes	Non‐Recommended Behaviour (Cross‐Cutting)	Experience of Functional and Emotional Consequences	Evaluate the perceived positive or negative impact of the deviation as a central outcome for future decisions.

*Note:* Cross‐cutting categories represent inductively derived elements identified across multiple symptom management domains.

Abbreviation: NRB, non‐recommended behaviour.

#### 3.3.3 Perceived Consequences of Symptom Management (Third Dimension of Dodd's Model)

Across the included studies, outcomes were not reported as formal endpoints. Instead, descriptive indications of perceived results appear indirectly within patients’ accounts. These perceived consequences relate to (a) perceived symptom control, (b) emotional and cognitive responses, and (c) consequences for daily functioning and participation. Evidence remains heterogeneous and context‐dependent, reflecting patient‐reported impressions rather than systematically evaluated effects.

##### 3.3.3.1 Perceived Symptom Control

Patients associated various symptom‐related actions with immediate short‐term relief. Specifically, perceived reductions in breathlessness and fatigue emerged as subjective consequences of symptom‐related actions [17, 28, 35, 41, 42, 45]. Furthermore, perceived early symptom recognition was linked to consistent monitoring routines. For those adhering to weight or oedema tracking, the perceived benefit lay in the ability to initiate timely diuretic adjustments in response to changes [18, 24, 39, 43].

In cases where patients modified medication based on their own experience, perceived stabilisation of fluid retention was described as transient [21, 28, 34, 38, 44].

Conversely, some deviations from recommended behaviour resulted in short‐term relief or practical advantages (e.g., adjusting medication to reduce side effects, relaxing dietary restrictions on social occasions) [19, 24, 32, 34, 42]. However, several studies also reported situations where delayed responses, inconsistent monitoring, or avoidance were followed by worsening symptoms or increased uncertainty [18, 21, 24, 27, 31, 33, 34, 39, 42, 44, 46]. Adjusting or discontinuing diuretics because of travel, workplace demands, or lack of restroom availability sometimes led to later symptom escalation requiring help‐seeking or compensation [17, 20, 21, 34, 44].

These accounts describe only perceived improvements from specific actions but do not reflect evaluated effectiveness.

##### 3.3.3.2 Emotional and Cognitive Outcomes

Patients frequently experienced emotional reactions in connection with symptom management strategies. Perceiving the successful application of strategies and behaviours resulting in stabilisation of symptoms, fostered a sense of self‐efficacy. Some found reassurance or an increased sense of control in involving family members or drawing on previous experiences of symptoms [17, 21, 22, 23, 28, 36, 43]. Support from relatives or healthcare providers was described as emotionally relieving when it helped clarify symptom meaning or encouraged appropriate action [17, 36]. At the same time, uncertainty remained common especially when symptoms were ambiguous or fluctuating [19, 21, 32, 41, 42]. Fear of deterioration, concerns about hospitalisation, or emotional overload contributed to avoidance or delayed responses, thereby increasing long‐term distress [17, 19, 24, 28, 32, 34, 42, 45]. If symptoms persisted despite self‐initiated measures, patients reported rising concern, prompting help‐seeking [26, 31, 37]. Overall, emotional outcomes influenced how patients interpreted symptoms and decided on subsequent actions.

##### 3.3.3.3 Daily Functioning and Social Participation

Patients reported that adaptations such as structuring activities, conserving energy, and pacing enabled them to maintain daily routines despite functional limitations [17, 21, 22, 27, 32, 35].

Use of mobility aids, household tools, and home modifications supported the continuation of daily tasks and increased safety [17, 22, 35, 45]. However, several studies indicated that symptom burden and behavioural recommendations (e.g., fluid restriction) sometimes limited social participation [20, 24, 42]. Avoidance behaviours due to breathlessness, fear of falls, or concerns about symptom exacerbation reduced mobility outside the home [17, 19, 24, 44, 45].

Some patients described withdrawing from social events to avoid pressure to drink or eat against recommendations [20, 24, 42]. Thus, outcomes ranged from maintained autonomy through adaptive strategies to reduced participation associated with symptom‐related constraints.

## 4 Discussion

The interpretation of the results is based on a refined analysis of Dodd's symptom management model [6], because it conceptualises symptom experience as a dynamic process shaped by the interaction of symptom perception, management strategies, and outcomes. Therefore, this framework provides a useful perspective for structuring the results of this study, as it takes into account both the subjective experiences of patients and the contextual factors that influence their responses. While the model offers a high structural fit for categorising CHF‐related phenomena, our synthesis suggests that its analytical value lies in emphasising the fluid transitions between dimensions rather than their conceptual separation. Furthermore, our findings support the applicability of Dodd's model for structuring symptom‐related processes in CHF. At the same time, the synthesis suggests the need for a more differentiated analytical emphasis on the interplay between symptom experience (D1), management strategies (D2), and perceived outcomes (D3), as enacted in patients’ everyday decision‐making.

A central finding concerns the dual nature of symptom monitoring. In line with the theory of Riegel et al. [48], symptom monitoring emerges as a dual‐function process operating across dimensions. While formally positioned as a management strategy (D2), it is already deeply embedded in symptom experience (D1) as an interpretive tool. Patients use symptom monitoring to translate vague bodily sensations into assessable indicators. Daily weight monitoring exemplifies this process: while technically a management strategy (D2), it primarily serves as an objective reference point that patients’ use to validate subjective perceptions such as dyspnoea (D1) and to decide whether active management is required [49, 50].

Our synthesis further suggests an alternative conceptualisation of NRB. Rather than interpreting deviations from recommendations primarily as a lack of adherence, NRB can be understood as a phenomenon with direct relevance for symptom outcomes (D3). Such behaviours often result from situationally rational trade‐offs, in which the preservation of quality of life and personal priorities outweigh strict guideline conformity. These processes of naturalistic decision‐making [48] aim to maintain perceived control over everyday life [51]. Positioning NRB at the interface between management strategies and outcomes (D2/D3) highlights that such decisions often reflect deliberate attempts to preserve autonomy under real‐life challenges. The determinants identified in this review were derived exclusively from studies focusing on NRB and are, therefore, conceptualised as factors contributing specifically to the emergence of NRB rather than as general contextual influences.

A third analytical layer concerns the role of social support and assistive tools as cross‐cutting enablers. The involvement of social networks and the use of practical aids (e.g., pill organisers or symptom diaries) modulate the entire management cycle by validating symptom perceptions in D1 and stabilising implementation in D2. Digital resources such as telemonitoring or eHealth applications [52, 53] strengthen the technological infrastructure of symptom management and support continuity of symptom monitoring. At the same time, access to and effective use of these resources are context‐dependent, underscoring the importance of considering social and structural inequalities when implementing such approaches.

Although further reciprocal relationships between the model dimensions are conceptually plausible, the available data did not allow these to be modelled explicitly within the present synthesis.

### 4.1 Implications for Clinical Practice

These findings point to important implications for clinical practice that require closer alignment with patient experience. As symptom experience (D1) and management (D2) are interlinked, clinical care should address both formal and informal support networks as integrated systems. Heart failure specialist nurses are well positioned to bridge the gap between patients’ everyday strategies and guideline‐oriented care [54, 55] by supporting both symptom perception (D1) and management execution (D2). Evidence indicates that nurse‐led counselling, structured follow‐up, and telecoaching programmes can reduce knowledge gaps, enhance self‐efficacy, and support timely help‐seeking behaviour [55, 56]. Their integration into routine care, with continuity and low‐threshold access, can help align treatment recommendations with patient‐reported needs [54, 56, 57]. This also implies that support must be context‐sensitive, taking into account patients’ individual symptom trajectories, everyday constraints, and available social resources. Rather than relying on uniform educational approaches, interventions should be adapted to patients’ lived realities and to the dynamic nature of their condition.

Simultaneously, the integration of family members is central. Patients frequently rely on relatives for validating initial symptoms (D1) as well as for medication routines, monitoring activities, and decision‐making (D2), effectively positioning them as co‐managers in everyday symptom management [58, 59]. Targeted inclusion of relatives in educational interventions may improve adherence and reduce uncertainty [60, 61], but also requires attention to caregiver burden, as caregiver involvement may increase stress in the absence of adequate support [61, 62]. Assistive tools (e.g., pill organisers, symptom diaries) and digital solutions represent promising complements for stabilising shared symptom management in daily life [52, 53].

Regarding symptom outcomes (D3), this review highlights that NRB often reflects attempts to preserve control and quality of life under everyday challenges [51]. Recognising such behaviours as adaptive strategies rather than mere non‐adherence opens opportunities for more constructive clinical dialogue. Approaches such as shared decision‐making [63] or motivational interviewing [64] may support clinicians in engaging with patients’ underlying reasoning and in co‐developing realistic and sustainable action plans.

### 4.2 Methodological Considerations

A notable methodological strength of this Scoping Review lies in its broad search strategy, which extended beyond the term “self‐management” to include “symptom management” and related concepts. This enabled the identification of studies that examined relevant sub‐aspects of symptom management, even when it was not their primary focus. As many of these studies addressed symptom management within broader health concepts, this approach allowed the capture of symptom‐related behaviours and strategies that might otherwise have been overlooked.

The review incorporated qualitative, quantitative and mixed‐methods primary studies. Qualitative studies provided in‐depth insight into decision‐making processes, while quantitative studies offered larger samples and standardised measurements, and mixed‐methods studies combined both strengths. This methodological diversity allowed for a broad and nuanced understanding but limited the comparability of findings across studies.

A further key step was the systematic distinction between symptom management and self‐management. Because many included studies framed symptom management as part of self‐management, a structured content analysis according to the Top‐Down‐Bottom‐Up approach by Mayring [16] was applied. Deductive categories were derived from current heart failure guidelines [4, 5, 8] and Dodd's model [6], while inductive categories captured previously unaddressed aspects. This ensured a clear delineation of symptom management while allowing new insights to emerge from the data.

### 4.3 Patient and Public Involvement (PPIE)

The PPIE contributor supported the plausibility and everyday resonance of the categories. They valued the micro‐level descriptions of strategies as practically actionable and endorsed framing deviations from recommendations as coping within real‐life constraints rather than mere non‐adherence, which aligned with our interpretation. They emphasised the importance of family involvement and low‐threshold access to advice, particularly via Heart Failure Specialist Nurses, and noted that such roles are often poorly recognised in routine care. Peer support also emerged as a desirable adjunct. These inputs refined the wording and emphases of the results and conclusions without altering the evidence base.

### 4.4 Future Research

Future studies should examine the mechanisms and outcomes of patient‐devised strategies, including NRBs, to understand their impact on long‐term disease trajectories. Evaluating approaches for integrating heart failure specialist nurses and digital tools into routine care, as well as studying effective forms of family involvement, could provide important evidence for implementing patient‐centred models of symptom management.

Although the review did not explicitly examine geographical differences, the included studies were predominantly conducted in high‐income countries. The paucity of evidence from low‐ and middle‐income countries (LMICs) is likely multifactorial, reflecting constraints in research infrastructure, limited access to specialised heart failure services, and publication and indexing patterns that favour high‐income settings. This gap restricts the transferability of current evidence to contexts where formal follow‐up, telemonitoring, and/or specialist nursing roles may be less available. Importantly, several mechanisms identified in this review, such as reliance on informal social support, low‐threshold monitoring practices, and adaptive, situational decision‐making, may be particularly salient under resource constraints. Future research should, therefore, prioritise (i) patient‐reported symptom management strategies in LMIC contexts, (ii) the role of social networks as co‐managers, and (iii) resource‐sensitive models of support that do not depend on high‐intensity infrastructure.

### 4.5 Limitations

Several methodological limitations should be considered when interpreting the findings of this review. The distinction between symptom management and self‐management was not always clearly possible due to the available literature. A high proportion of studies treated symptom management as part of the broader concept of self‐management, making the distinction methodologically challenging. To address this challenge, a structured content analysis according to Mayring was employed [16]. Nevertheless, it is acknowledged that alternative theoretical models or an exclusively inductive approach might have resulted in a different categorisation. In addition, it cannot be ruled out that relevant publications were missed despite the comprehensive search strategy, since symptom management is frequently incorporated into broader conceptual frameworks and may not be explicitly labelled. A further limitation concerns the geographical distribution of the included studies. The majority of the studies were conducted in the USA and Europe, while studies from other regions such as Asia, Australia, or Africa were underrepresented. This may limit the generalisability of the findings to other cultural or healthcare system contexts. Although NYHA classification was reported in 23 studies (with most participants classified as Class II or III), symptom management strategies could not be differentiated by NYHA class due to inconsistent reporting across studies. Therefore, our synthesis more broadly reflects behaviours across the CHF population. As no formal quality appraisal was undertaken, these findings represent a mapping of existing evidence rather than a quality‐based evaluation. However, heterogeneity in study quality should be considered when interpreting the findings. Finally, PPIE involvement was limited to a single retrospective consultation. Nevertheless, it improved interpretability and practice relevance by corroborating category plausibility and refining emphasis areas.

## 5 Conclusion

Symptom management in people with CHF is a complex and ongoing challenge. This review shows that patients use diverse strategies to monitor and manage their symptoms, shaped by their subjective perceptions, social resources, and everyday constraints. Some strategies align closely with guideline‐based care, while others deviate from recommendations as patients strive to maintain autonomy, stability, and quality of life. Interpreting such behaviours as contextually adaptive rather than as simple non‐adherence provides a more patient‐centred understanding of symptom management and may facilitate more constructive clinical dialogue. Social support emerged as a central factor for implementing and sustaining symptom management. Family members act as co‐managers in daily care, and their involvement can strengthen patients’ trust in treatment recommendations while reducing uncertainty. Heart Failure Specialist Nurses are well positioned to translate recommendations into everyday practice by supporting personalised strategies, counselling, and education. Dodd's Symptom Management Model offers a valuable framework for conceptualising the interrelations between symptom experience, management strategies, and perceived outcomes, and for guiding the development of patient‐centred approaches to symptom management in CHF.

## Author Contributions

**Severin Pietsch:** conceptualisation, methodology, study design, visualization, literature search, screening and selection – articles, data extraction and analysis, and writing – manuscript. **Sascha Köpke** and **Christa Büker:** supervision, writing – review and editing. **Irene Müller:** screening and selection – article, data extraction, data validation, writing – review and editing. All authors: review and approval – final manuscript.

## Ethics Statement

This study did not require ethical approval as it is based on previously published data.

## Conflicts of Interest

The authors declare no conflicts of interest.

## Supporting information

Supporting File

## Data Availability

Data sharing not applicable to this article as no datasets were generated or analysed during the current study.
