# Supplementary material for: Symptom Management in Chronic Heart Failure: Strategies and Behaviours From Patients’ Perspectives—A Scoping Review
Source: Health Expect. 2026 Apr 13;29(2):e70667. doi: 10.1111/hex.70667 (PMC13074360; doi:10.1111/hex.70667)
Supplement: Supplementary file 1 — Supporting File [file HEX-29-e70667-s001.docx]

Supplementary Table S1 Overview of studies excluded after full-text review, including the stated reasons for exclusion according to eligibility criteria.

Table S1. Overview of studies excluded after full-text review, including the stated reasons for exclusion according to eligibility criteria.

| No. | Excluded report | Reason for exclusion |
| --- | --- | --- |
| 1 | Buck HG, Harkness K, Wion R, Carroll SL, Cosman T, Kaasalainen S, et al. Caregivers' contributions to heart failure self-care: a systematic review. Eur J Cardiovasc Nurs. 2015;14(1):79-89. doi:10.1177/1474515113518434 | Relatives’ perspectives on behaviours and strategies |
| 2 | Cavalcante AMRZ, Lopes CT, Brunori EFR, Swanson E, Moorhead SA, Bachion MM, et al. Self-care behaviors in heart failure. Int J Nurs Knowl. 2018;29(3):146-55. doi:10.1111/2047-3095.12170 | No specific behaviours and strategies described |
| 3 | Checa C, Medina-Perucha L, Muñoz MÁ, Verdú-Rotellar JM, Berenguera A. Living with advanced heart failure: a qualitative study. PLoS One. 2020;15(12):e0243974. doi:10.1371/journal.pone.0243974 | No specific behaviours and strategies described |
| 4 | Cavalcante LM, Lima FET, Custódio IL, Oliveira SKP, Meneses LST, Oliveira ASS, et al. Influence of socio-demographic characteristics in the self-care of people with heart failure. Rev Bras Enferm. 2018;71(Suppl 6):2604-11. doi:10.1590/0034-7167-2017-0480 | No specific behaviours and strategies described |
| 5 | Chung ML, Lennie TA, de Jong M, Wu JR, Riegel B, Moser DK. Patients differ in their ability to self-monitor adherence to a low-sodium diet versus medication. J Card Fail. 2008;14(2):114-20. doi:10.1016/j.cardfail.2007.10.010 | No specific behaviours and strategies described |
| 6 | Daley CN, Cornet VP, Toscos TR, Bolchini DP, Mirro MJ, Holden RJ. Naturalistic decision making in everyday self-care among older adults with heart failure. J Cardiovasc Nurs. 2022;37(2):167-76. doi:10.1097/JCN.0000000000000778 | No specific behaviours and strategies described |
| 7 | Dickson VV, Buck H, Riegel B. A qualitative meta-analysis of heart failure self-care practices among individuals with multiple comorbid conditions. J Card Fail. 2011;17(5):413–9. doi:10.1016/j.cardfail.2010.11.011 | Ineligible study design (systematic review) |
| 8 | Dickens C, Dickson VV, Piano MR. Perceived stress among patients with heart failure who have low socioeconomic status: a mixed-methods study. J Cardiovasc Nurs. 2019;34(3):E1-8. doi:10.1097/JCN.0000000000000562 | No specific behaviours and strategies described |
| 9 | Harkness K, Spaling MA, Currie K, Strachan PH, Clark AM. A systematic review of patient heart failure self-care strategies. J Cardiovasc Nurs. 2015;30(2):121–35. doi:10.1097/JCN.0000000000000118 | Ineligible study design (systematic review) |
| 10 | Jeon YH, Kraus SG, Jowsey T, Glasgow NJ. The experience of living with chronic heart failure: a narrative review of qualitative studies. BMC Health Serv Res. 2010;10:77. doi:10.1186/1472-6963-10-77 | Ineligible study design (systematic review) |
| 11 | Kim DY, Son YJ. Longitudinal patterns and predictors of self-care behavior trajectories among Korean patients with heart failure: a 6-month prospective study. J Nurs Scholarsh. 2023;55(2):429-38. doi:10.1111/jnu.12833 | No specific behaviours and strategies described |
| 12 | Lam C, Smeltzer SC. Patterns of symptom recognition, interpretation, and response in heart failure patients: an integrative review. J Cardiovasc Nurs. 2013;28(4):348-59. doi:10.1097/JCN.0b013e3182531cf7 | No specific behaviours and strategies described |
| 13 | Lee CS, Bidwell JT, Paturzo M, Alvaro R, Cocchieri A, Jaarsma T, et al. Patterns of self-care and clinical events in a cohort of adults with heart failure: 1 year follow-up. Heart Lung. 2018;47(1):40-46. doi:10.1016/j.hrtlng.2017.09.004 | No specific behaviours and strategies described |
| 14 | Meraz R. Medication nonadherence or self-care? Understanding the medication decision-making process and experiences of older adults with heart failure. J Cardiovasc Nurs. 2020;35(1):26-34. doi:10.1097/JCN.0000000000000616 | No specific behaviours and strategies described |
| 15 | Nordfonn OK, Morken IM, Bru LE, Husebø AML. Patients' experience with heart failure treatment and self-care: a qualitative study exploring the burden of treatment. J Clin Nurs. 2019;28(9-10):1782-93. doi:10.1111/jocn.14799 | No specific behaviours and strategies described |
| 16 | Riegel B, Dickson VV, Topaz M. Qualitative analysis of naturalistic decision making in adults with chronic heart failure. Nurs Res. 2013;62(2):91-8. doi:10.1097/NNR.0b013e318276250c | No specific behaviours and strategies described |
| 17 | Graven LJ, Grant JS. Social support and self-care behaviors in individuals with heart failure: an integrative review. Int J Nurs Stud. 2014;51(2):320-33. doi:10.1016/j.ijnurstu.2013.06.013 | No specific behaviours and strategies described |
| 18 | Halmo R, Galuszka J, Langova K, Galuszkova D. Self care in patients with chronic heart failure. Pilot study – self care includes problems. Biomed Pap Med Fac Univ Palacky Olomouc Czech Repub. 2015;159(1):124-30. doi:10.5507/bp.2013.044 | No specific behaviours and strategies described |
| 19 | Holden RJ, Schubert CC, Eiland EC, Storrow AB, Miller KF, Collins SP. Self-care barriers reported by emergency department patients with acute heart failure: a sociotechnical systems-based approach. Ann Emerg Med. 2015;66(1):1-12.e2. doi:10.1016/j.annemergmed.2014.12.031 | No specific behaviours and strategies described |
| 20 | Jones CD, Holmes GM, DeWalt DA, Erman B, Wu JR, Cene CW, et al. Self-reported recall and daily diary-recorded measures of weight monitoring adherence: associations with heart failure-related hospitalization. BMC Cardiovasc Disord. 2014;14:12. doi:10.1186/1471-2261-14-12 | No specific behaviours and strategies described |
| 21 | Kamath DY, Bhuvana KB, Salazar LJ, Varghese K, Kamath A, Idiculla J, et al. A qualitative, grounded theory exploration of the determinants of self-care behavior among Indian patients with a lived experience of chronic heart failure. PLoS One. | No specific behaviours and strategies described |
| 22 | Lee KS, Oh S. An integrative review of the symptom perception process in heart failure. J Cardiovasc Nurs. 2022;37(2):122-33. doi:10.1097/JCN.0000000000000750 | No specific behaviours and strategies described |
| 23 | Oguz S, Enç N. Symptoms and strategies in heart failure self-management in Turkey [corrected]. Int Nurs Rev. 2008;55(4):462-7. doi:10.1111/j.1466-7657.2008.00661.x | No specific behaviours and strategies described |
| 24 | Mapelli M, Salvioni E, Bonomi A, Gugliandolo P, De Martino F, Vignati C, et al. How patients with heart failure perform daily life activities: an innate energy-saving strategy. Circ Heart Fail. 2020;13(11):e007503. doi:10.1161/CIRCHEARTFAILURE.120.007503 | No specific behaviours and strategies described |
| 25 | Min D, Lee J, Ahn JA. A qualitative study on the self-care experiences of people with heart failure. West J Nurs Res. 2023;45(7):646-52. doi:10.1177/01939459231169102 | No specific behaviours and strategies described |
| 26 | Rababah JA, Al-Hammouri MM, Tawalbeh L, Alhawatmeh H, Hasan M. Self-care among persons with heart failure: examining the impact of mindfulness and impulsivity using conditional process analysis. Nurs Forum. 2022;57(4):558-67. doi:10.1111/nuf.12719 | No specific behaviours and strategies described |
| 27 | Reeder KM, Peek GM, Nazir N. Prehospitalization symptom perceptions, lay consultations, and treatment-seeking for acute decompensating heart failure: implications for nursing practice. Crit Care Nurs Clin North Am. 2022;34(2):129-40. doi:10.1016/j.cnc.2022.02.002 | No specific behaviours and strategies described |
| 28 | 25. Riegel B, Vaughan Dickson V, Goldberg LR, Deatrick JA. Factors associated with the development of expertise in heart failure self-care. Nurs Res. 2007;56(4):235-43. doi:10.1097/01.NNR.0000280615.75447.f7 | No specific behaviours and strategies described |
| 29 | Sayers SL, Riegel B, Pawlowski S, Coyne JC, Samaha FF. Social support and self-care of patients with heart failure. Ann Behav Med. 2008;35(1):70-9. doi:10.1007/s12160-007-9003-x | No specific behaviours and strategies described |
| 30 | Schjoedt I, Sommer I, Bjerrum MB. Experiences and management of fatigue in everyday life among adult patients living with heart failure: a systematic review of qualitative evidence. JBI Database Syst Rev Implement Rep. 2016;14(3):68-115. doi:10.11124/JBISRIR-2016-2441 | No specific behaviours and strategies described |
| 31 | Seid MA, Abdela OA, Zeleke EG. Adherence to self-care recommendations and associated factors among adult heart failure patients: from the patients' point of view. PLoS One. 2019;14(2):e0211768. doi:10.1371/journal.pone.0211768 | No specific behaviours and strategies described |
| 32 | Song EK, Moser DK, Kang SM, Lennie TA. Self-reported adherence to a low-sodium diet and health outcomes in patients with heart failure. J Cardiovasc Nurs. 2016;31(6):529-34. doi:10.1097/JCN.0000000000000287 | No specific behaviours and strategies described |
| 33 | Sousa JP, Oliveira C, Pais-Vieira M. Symptom perception management education improves self-care in patients with heart failure. Work. 2021;69(2):465-73. doi:10.3233/WOR-213491 | No specific behaviours and strategies described |
| 34 | Sousa JP, Santos M. Symptom management and hospital readmission in heart failure patients: a qualitative study from Portugal. Crit Care Nurs Q. 2019;42(1):81-88. doi:10.1097/CNQ.0000000000000241 | No specific behaviours and strategies described |
| 35 | Spaling MA, Currie K, Strachan PH, Harkness K, Clark AM. Improving support for heart failure patients: a systematic review to understand patients' perspectives on self-care. J Adv Nurs. 2015;71(11):2478-89. doi:10.1111/jan.12712 | No specific behaviours and strategies described |
| 36 | Tawalbeh LI, Al-Smadi AM, AlBashtawy M, AlJezawi M, Jarrah M, Musa AS, et al. The most and the least performed self-care behaviors among patients with heart failure in Jordan. Clin Nurs Res. 2020;29(2):108-16. doi:10.1177/1054773818779492 | No specific behaviours and strategies described |
| 37 | Vanstone M, Giacomini M, Smith A, Brundisini F, DeJean D, Winsor S. How diet modification challenges are magnified in vulnerable or marginalized people with diabetes and heart disease: a systematic review and qualitative meta-synthesis. Ont Health Technol Assess Ser. 2013;13(14):1-40. | No specific behaviours and strategies described |
| 38 | Walthall H, Floegel T. The lived experience of breathlessness for people diagnosed with heart failure: a qualitative synthesis of the literature. Curr Opin Support Palliat Care. 2019;13(1):18-23. doi:10.1097/SPC.0000000000000405 | No specific behaviours and strategies described |
| 39 | White MM, Howie-Esquivel J, Caldwell MA. Improving heart failure symptom recognition: a diary analysis. J Cardiovasc Nurs. 2010;25(1):7-12. doi:10.1097/JCN.0b013e3181b7af9e | No specific behaviours and strategies described |
| 40 | Wiśnicka A, Lomper K, Uchmanowicz I. Self-care and quality of life among men with chronic heart failure. Front Public Health. 2022;10:942305. doi:10.3389/fpubh.2022.942305 | No specific behaviours and strategies described |
